# FAnet: A lightweight end-to-end framework for Fructus Aurantii identification and quality grading

**DOI:** 10.1371/journal.pone.0357781

**Published:** 2026-09-28

**Authors:** LeTian Wu, KeKe Liao, BingWei Song, ShenBo Guo, XingDong Gao, HuiFeng Shi

**Affiliations:** 1 Institute of Agricultural Equipment, Xinjiang Uygur Autonomous Region Academy of Agricultural Sciences, Urumqi, China; 2 Xinjiang Key Laboratory of Intelligent Control Technology for Facility Agriculture, Urumqi, China; 3 College of Engineering in Jiangxi Agricultural University, Jiangxi Provincial Key Laboratory of Modern Agricultural Equipment, Nanchang, China; Apeejay Stya University, INDIA

## Abstract

The quality grade of Fructus Aurantii(FA) is closely related to its clinical efficacy and medication safety; therefore, a standardized grading method is needed. However, FA samples show considerable morphological variation, and conventional grading depends largely on manual visual assessment, which is subjective and inefficient. This study proposes FAnet, a lightweight instance segmentation framework based on YOLOv8n-seg, for estimating peel thickness and outer diameter during FA quality grading. FAnet integrates three task-adapted components: C2Star, which combines StarNet and C2f to improve multi-scale feature extraction; the Simple Parameter-Free Attention Module (SimAM), which enhances spatial-channel feature weighting without introducing additional trainable parameters; and a multi-scale dynamic fitting module (MSDFM), which maps segmentation-derived areas to physical dimensions for grading. Using a self-built dataset containing 1,093 original images and 5,035 augmented training images, FAnet achieved 96.12% precision, 98.77% recall, 98.60% mAP@50, and 77.08% mAP@95, with 2.97 M parameters and 7.9 GFLOPs under the present experimental setting. The results indicate that FAnet can support automated FA segmentation and preliminary grading under controlled imaging conditions. Broader validation across larger and more diverse sample populations is still required before deployment-oriented application.

## Introduction

Fructus Aurantii (FA), commonly known as Zhiqiao, is a traditional Chinese medicine recorded in the Chinese Pharmacopoeia. It is used for regulating Qi, reducing abdominal distension, and promoting digestion [1]. FA quality affects clinical efficacy and medication safety. Conventional identification and grading rely on manual evaluation of morphological characteristics, color, and texture by experienced personnel. These approaches are limited by low efficiency, high subjectivity, and insufficient standardization. In recent years, machine-vision-based intelligent detection methods have been increasingly applied to agricultural product sorting and industrial quality inspection, offering a potential route for automated identification and grading of traditional Chinese medicines. However, FA has complex surface texture, variable morphology, and background interference, which can reduce feature extraction accuracy, model generalization, and computational efficiency. Therefore, an automated FA grading method needs to balance accuracy, robustness, and computational cost [2].

In recent years, deep learning technology has introduced a new technical paradigm for the intelligent detection and grading of Chinese herbal medicines. In target detection, methods based on convolutional neural networks have demonstrated notable advantages. Chang et al. (2024) applied an improved ResNet50-based architecture to achieve precise localization of herbal materials [3]. Zhou et al. (2024) developed a hybrid model combining RGB, HOG, and LBP features with AlexNet for herbal classification [4]. However, the manual features and heterogeneous deep feature fusion introduced increased inference delays. Liu et al. (2021) applied the SSD algorithm and reported an accuracy of 92.7%; however, limitations in detecting small objects were observed [5]. In fine grained classification, Wang et al. (2020) reported a 5.3% improvement in classification accuracy using a multichannel color space attention mechanism [6]. Zhuang et al. (2018) developed a deep convolutional network to recognize 98 herbal medicine types, reaching 95.8% accuracy [7]. Nonetheless, the model’s large size (320M parameters) limits its deployment on edge devices.

The YOLO model series has become prominent due to efficiency and flexibility. Dong et al. (2023) added Coordinate Attention (CA) and a lightweight GhostNet module to YOLOv5s, reducing computation by 37% but decreasing detection recall for overlapping herbs by 9.2% [8]. Tao et al. (2023) introduced a Feature Enhancement Module (FEM) to recalibrate multi-scale features, mitigating information loss from small targets and increasing the mAP of overlapping licorice samples by 6.5% [9]. Li et al. (2023) introduced a redundant detection head, which reduced false detections but also decreased sensitivity to large-scale objects [10]. Luo et al. (2025) integrated Content Aware ReAssembly of Features (CARAFE) up-sampling and Multimatrix Factorization Attention (MFA) into YOLOv8 for Tibetan medicinal herb detection, achieving 89.4% mAP but increasing 23% computational overhead [11].

In segmentation and lightweight research, Tan et al. (2024) embedded Convolutional Block Attention Module (CBAM) in U-Net, attaining 86.2% IoU in Sichuan safflower semantic segmentation [12]. Feng et al. (2024) improved YOLOv8 by replacing standard convolutions with FasterNet’s PConv, reducing parameters by 21% and increasing maturity recognition accuracy of yellow essence seeds [13]. Deng et al. (2023) developed an ECANet dual-path fusion model based on Inception-ResNet-v2 with MSRCR-CLAHE image enhancement, achieving a 97.1% recognition rate for Angelica leaf spot disease [14]. Despite substantial advancements, persistent challenges remain. First, rigid multi-scale feature fusion mechanisms inadequately adapt to nonlinear characteristics of FA, particularly varying peel thickness. Second, balancing lightweight design with accuracy remains challenging. Third, morphological modeling is insufficient, and quantitative compensation strategies addressing peel texture deformation due to placement angles in static images are lacking.

In response to these challenges, this study proposes FAnet, an intelligent identification and grading framework tailored to FA. The framework integrates fusion feature extraction, attention enhancement, and multi-scale dynamic fitting to improve segmentation accuracy and grading efficiency. First, YOLOv8n-seg is used as the baseline model for rapid localization and instance segmentation of FA targets. Second, the C2f module is adapted by integrating StarNet operations to form C2Star, thereby improving the representation of fine surface and contour features. Third, SimAM is introduced to enhance feature weighting while maintaining a lightweight structure. Finally, the MSDFM is used as a post-processing module to establish a nonlinear mapping between pixel-level measurements and real-world geometric attributes, enabling quantitative grading based on estimated outer diameter and peel thickness. The main contribution of this study is the task-specific integration and validation of these components for FA grading, rather than the proposal of a general-purpose segmentation architecture.

## Materials

### Data collection

In this study, FA samples were dried immature fruits of the cultivated stinking orange variant Fructus Aurantii ‘Xiucheng’ (Rutaceae). All samples were collected in July 2024 from the Chinese Herbal Medicine Plantation Specialized Cooperative of Guangde Shuangkeng Village, Zhangshu City, Jiangxi Province, China (115.193551623 degrees E, 27.966390045 degrees N). The samples were dried by sun drying and oven drying [15].

During image acquisition, a tripod was used to maintain a fixed camera-to-object distance of 15 cm. The outer diameter of each FA sample was measured using a vernier caliper. Specifically, one equatorial diameter was selected as the initial reference, and four diameters were measured successively by rotating the sample by 45 degrees each time. The arithmetic mean of the four measurements was used as the final transverse diameter of the sample.

### Data calibration

Labelme, an open-source annotation tool, was used as the primary annotation platform and was combined with the Segment Anything Model (SAM) proposed by Meta AI for semi-automatic annotation [16,17]. The annotation target was the cross-sectional image of FA, including two key morphological features associated with grading requirements (Fig 1). The first feature was the inner flesh region, which mainly consisted of white spongy tissue separated by irregular polygonal boundaries between locules. The second feature was the outer peel contour, representing the external boundary of the fruit. During annotation, fruit stalks and shadowed regions were excluded where necessary so that the complete circular or oval contour was retained. All annotations were manually generated and then reviewed for quality control. Inner and outer boundaries were visually inspected and corrected, particularly in difficult cases with weak or ambiguous inner-boundary regions. Consistency checks were performed before model training to reduce labeling uncertainty.

**Fig 1:**
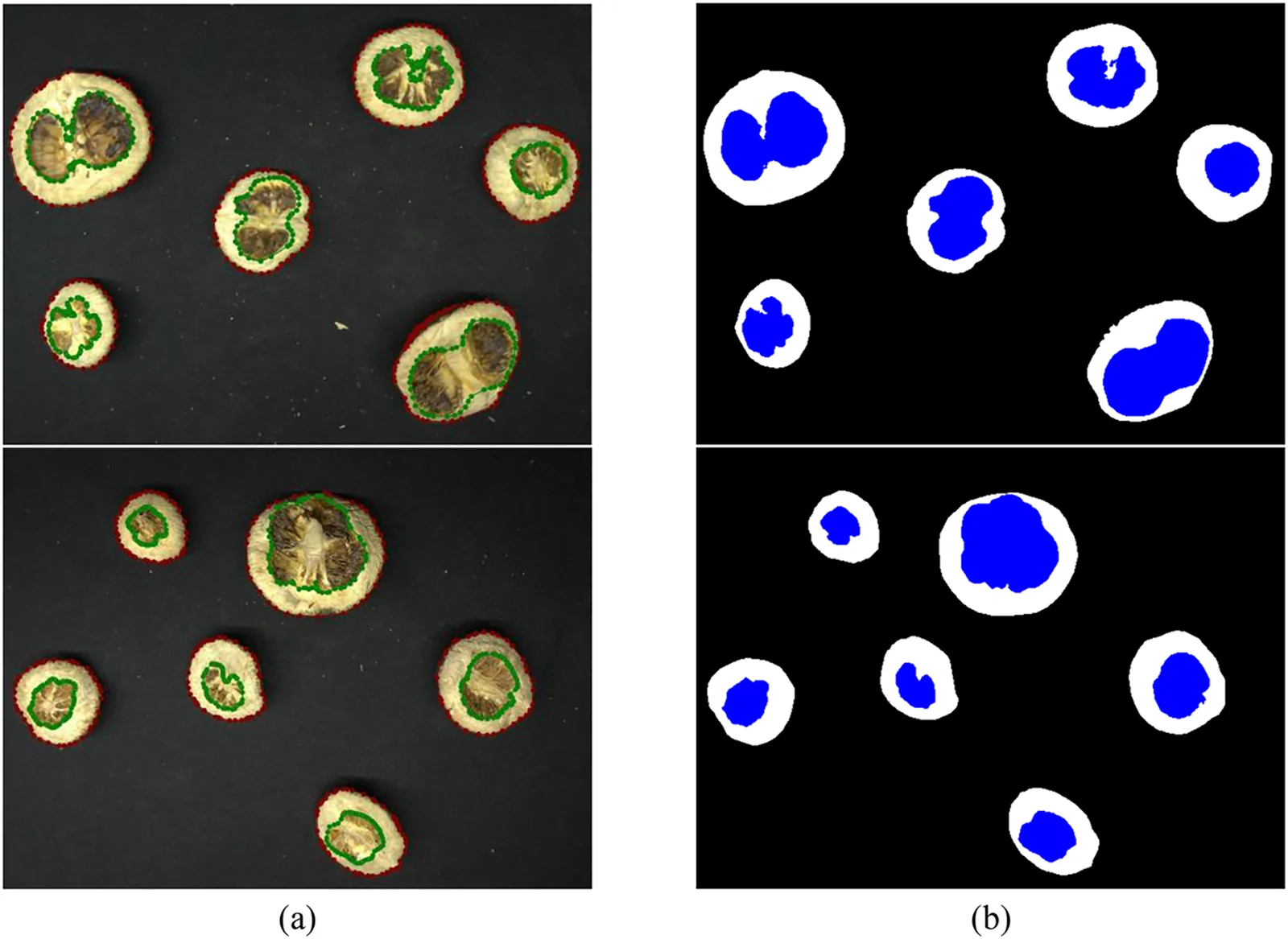
Fructus Aurantii image data: (a) AI labeling; (b) Label fusion.

### Data enhancement

To improve model adaptability to real-world image acquisition, this study used an algorithm-based augmentation strategy designed for FA images captured by mobile phones and cameras. The operations included random rotation within +/-25 degrees, horizontal flipping with a probability of 50%, random brightness and saturation adjustment in the HSV color space (+/-15% and +/-10%, respectively), color-temperature shifting within +/-500 K to simulate warm and cool illumination, generation of semi-transparent bright spots using Gaussian filtering (5–15 pixels, 30–60% transparency), random directional motion blur (5–15 pixels, 0–360 degrees), and ISO 800–3200 equivalent Poisson-Gaussian mixed noise [18]. Representative augmented images are shown in Fig 2, and the final dataset sizes are presented in **Table 1**. To prevent data leakage, all augmentation procedures were conducted only after the original train/validation/test split. Augmented images were generated exclusively from training data and were not used in validation or testing.

**Fig 2:**
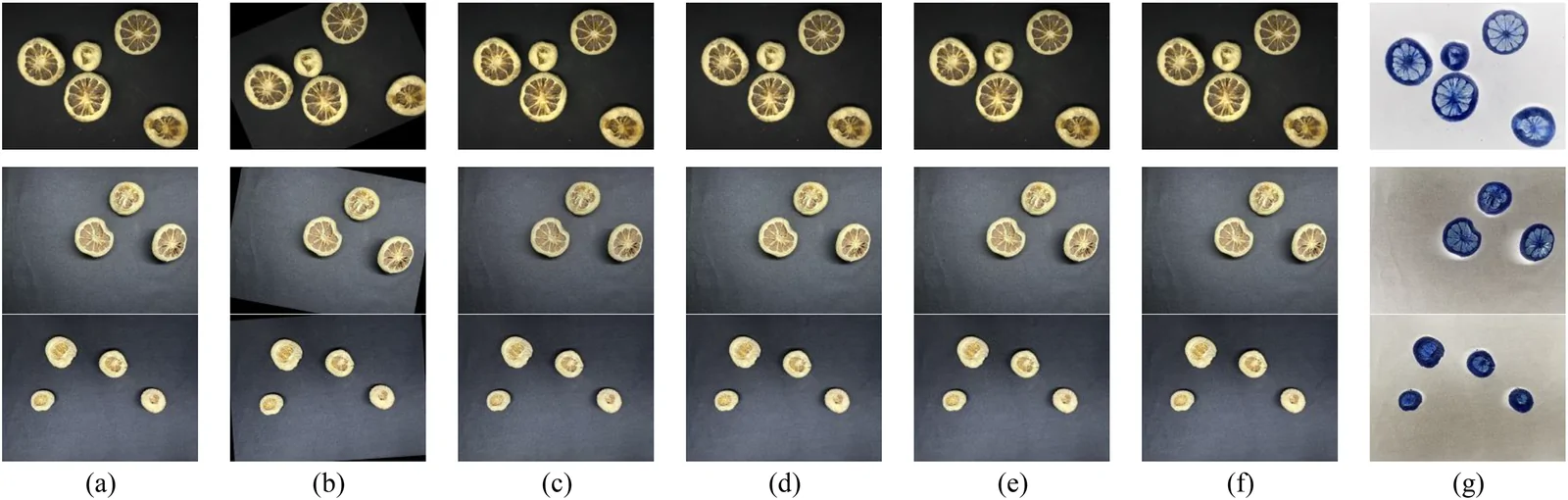
Images enhancement: (a) Original images; (b) Random flip; (c) Brightness and saturation adjustment; (d) Color temperature shift; (e) Gaussian filter bright spot; (f) random shift blur; (g) Poisson-Gaussian mixed noise.

**Table 1:** Data set count.

	Image number	Train set	Validation set	Test set
Original images	1093	657	218	218
Images enhancement	5035	4599	218	218

The dataset was split into training, validation, and test subsets prior to data augmentation. Augmentation operations were applied only to images in the training subset, and no augmented or near-duplicate versions of any source image appeared across validation or test subsets.

In addition, dataset partitioning was performed at the physical-sample level rather than the image level. Multiple images derived from the same Fructus Aurantii sample or from the same acquisition session were assigned exclusively to a single subset, thereby avoiding information leakage across subsets.

## Method

### Overall architecture of FAnet

To ensure clarity and reproducibility, FAnet is defined as a unified framework based on YOLOv8n-seg. The final architecture incorporates three task-adapted components: the C2Star module, the SimAM attention mechanism, and the multi-scale dynamic fitting module (MSDFM).

The baseline model adopts the anchor-free YOLOv8n-seg architecture, which integrates object detection and instance segmentation into a unified pipeline. The mask generation mechanism follows a prototype-based strategy similar to YOLACT, but no additional YOLACT structural components are introduced [19,20]. Within this framework, the modifications are applied consistently as follows: (1) the original C2f modules in the backbone and neck are replaced by C2Star modules to enhance multi-scale feature extraction through implicit high-dimensional feature expansion [21]; (2) SimAM is inserted at three fixed locations, namely after each C2Star module in the backbone, after the feature fusion outputs of the feature pyramid network (FPN), and before the segmentation head [22,23]; and (3) MSDFM is used as a post-processing module that maps normalized pixel areas of the inner and outer regions to real-world dimensions through polynomial regression. Peel thickness is then calculated from the predicted diameters. The final architecture is illustrated in Fig 3.

**Fig 3:**
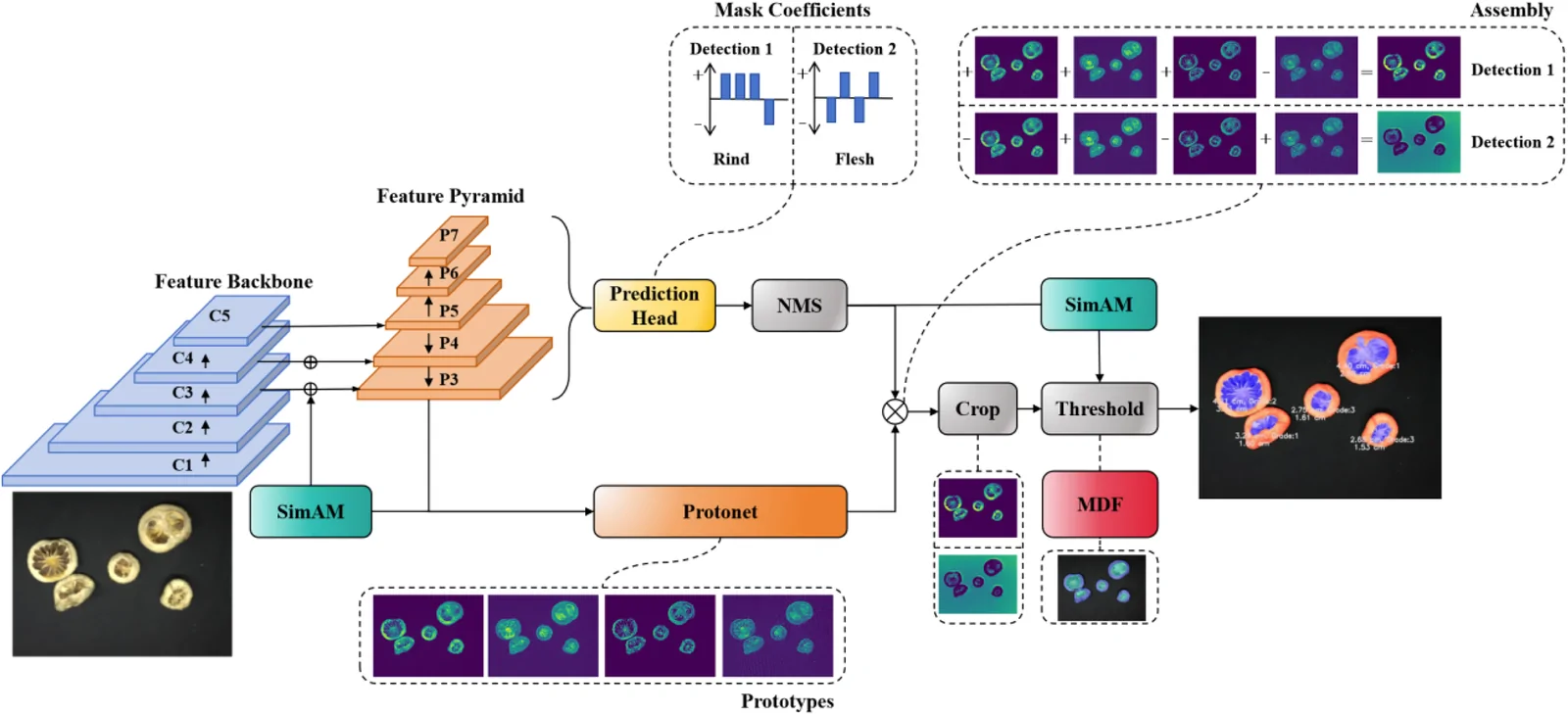
FAnet structure.

### Fusion feature extraction module C2Star

To improve the representation of fine-grained morphological features such as peel thickness and contour variation, this study uses C2Star as a replacement for the standard C2f module.

The C2Star module integrates the StarNet mechanism into the CSP-based structure. Specifically, Bottleneck blocks in C2f are replaced by Star blocks, which use element-wise multiplicative operations for nonlinear feature transformation. This design implicitly expands feature dimensionality without introducing substantial additional parameters.

Through recursive star operations, the module maps input features into a higher-dimensional implicit space, thereby improving the representation of subtle texture and structural variation. Compared with conventional convolution-based feature fusion, C2Star increases feature diversity while reducing computational redundancy.

Addressing the question of how to accurately identify subtle variations in FA peel thickness and surface texture, this research proposes the fused feature extraction module C2Star. Accurate detection of such small morphological differences requires networks capable of effectively capturing nuanced local features. While the original YOLOv8n-seg model employs the C2f module (derived from CSPDarknet53) to fuse low level and high level feature maps, thereby improving multi-scale detection accuracy. However, it greatly increases the computational complexity and prolongs the training time and computational resource requirements. To overcome these limitations, this research introduces the StarNet module into the C2Star structure. StarNet utilises star operations (element multiplication) for computational process optimisation. Through nonlinear feature transformation and extended implicit feature dimensions enhanced texture characterisation. Finally, achieving layer-by-layer extraction of FA features.

The C2Star structure is illustrated in Fig 4. The Bottleneck in C2f is to be replaced with Star Blocks, which are then to be combined to form a new module, C2Star, replacing the original C2f module [24]. Single level star operation for a d-dimensional input feature $x\in {\mathbb{R}}^{(d+1)\times n}$ and weight $\omega_{\text{1}},\omega_{\text{2}}\in {\mathbb{R}}^{\left(d+1)\times (d^{\prime }+1\right)}$, the star operation can be written as $\left(\omega_{\text{1}}^{T}x+B_{\text{1}})*(\omega_{\text{2}}^{T}x+B_{\text{2}}\right)$. Then its simplified to $\omega_{\text{1}}^{T}x*\omega_{\text{1}}^{T}x$. The feature is mapped from d-dimension to a higher dimension d′.

**Fig 4:**
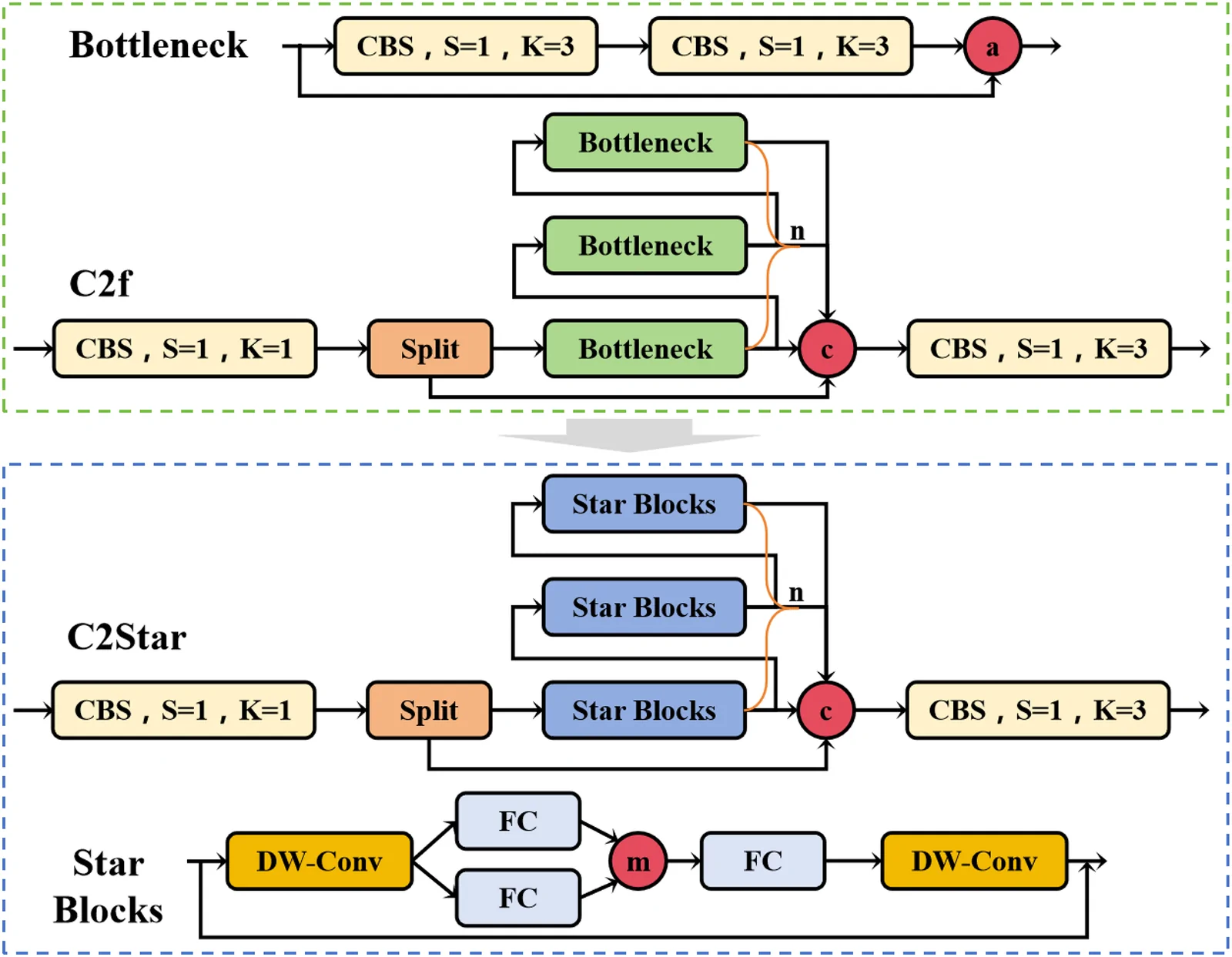
C2Star Fusion Structure.

The rewritten star operation formulas are shown in Eqs (1),(2),(3).


$$\begin{array}{c} \omega_{\text{1}}^{T}x*\omega_{\text{1}}^{T}x=\left(\sum_{i=1}^{d+1}\omega_{\text{1}}^{i}x^{i}\right)*\left(\sum_{j=1}^{d+1}\omega_{\text{1}}^{j}x^{j}\right) \end{array}$$ (1)

$$\begin{array}{c} \omega_{\text{1}}^{T}x*\omega_{\text{1}}^{T}x=\sum_{i=1}^{d+1}\sum_{j=1}^{d+1}\omega_{\text{1}}^{i}\omega_{\text{1}}^{j}x^{i}x^{j} \end{array}$$ (2)

$$\begin{array}{c} \omega_{\text{1}}^{T}x*\omega_{\text{1}}^{T}x=\underset{(d+2)(d+1)/2\begin{array}{cc} & items \end{array}}{\underbrace{\alpha_{\left(1,1\right)}x^{\text{1}}x^{\text{1}}+\cdots +\alpha_{\left(4,5\right)}x^{\text{4}}x^{\text{5}}+\cdots +\alpha_{\left(d+1,d+1\right)}x^{d+1}x^{d+1}}} \end{array}$$ (3)

In the above equation, when d>> 2, the rewrite operation maps the d-dimensional features to the $\frac{\left(d+2)(d+1\right)}{\text{2}}\approx (\frac{d}{\sqrt{\text{2}}}{)}^{\text{2}}$ dimensional implicit feature space, exponentially increasing the implicit dimensions in a recursive manner, which amplifies the feature dimensions without incurring computational overheads outside of the frontal x within a single layer. In Eq (3), each term except $\alpha_{\left(d+1,d+1\right)}x^{d+1}x^{d+1}$ is nonlinearly associated with x, indicating that they are independent, implicit dimensions [25].

### Integration of SimAM lightweight attention module

When traditional vision methods deal with FA features, there are low contrast features between the rind edge and the flesh and nonlinear spatial variability in rind thickness. They often need to rely on complex multi-scale filtering. They also need morphological preprocessing processes. These lead to an exponential increase in computational complexity. As a result, it becomes difficult to meet the demand for efficient lightweight detection. In this research, we propose to embed SimAM (Simple Parameter Free Attention Module) 3D attention mechanism in YOLOv8n-seg (Fig 5) [26]. The modeling is done through the null domain channel synergetic attention without parameterization and reduces computational and storage overhead. The mean and variance of the feature map itself are directly utilized to construct the energy function. Feature calibration is accomplished in a single forward propagation. Reduce the iterative computation than the traditional method.

**Fig 5:**
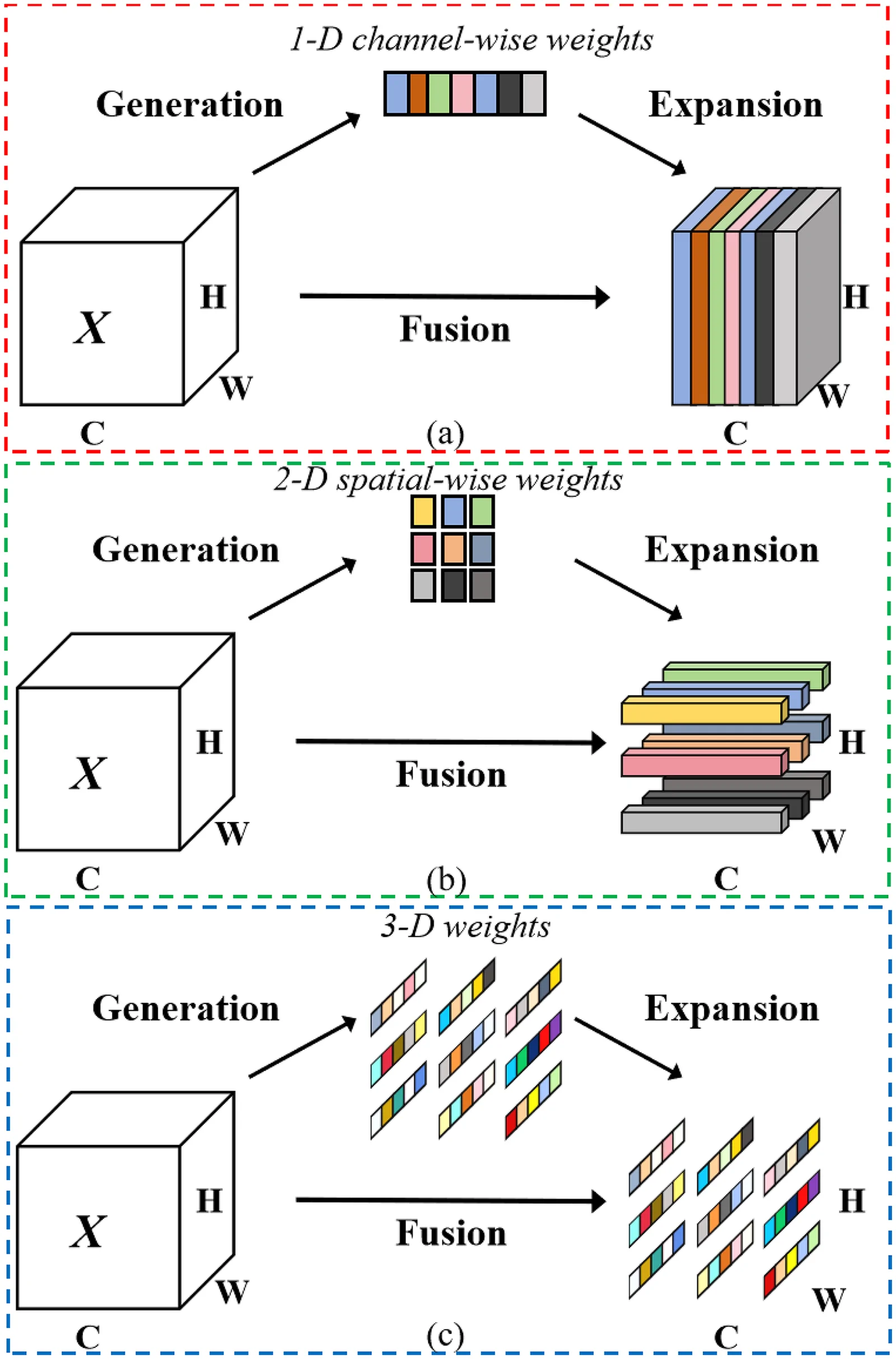
SimAM Lightweight Attention Module: (a) Channel-wise attention; (b) Spatial-wise attention; (c) Full 3-Dweights for attention.

The mechanism synchronizes feature enhancement and computational optimization by strengthening the high frequency response of the rind thickness mutation region. Existing channel or spatial attention modules are different. The SimAM requires no additional parameters. It can adaptively learn similarity information between targets. Also, it can weight the importance of the adjusted features. To better realize attention, the importance of each neuron needs to be evaluated. In neural networks, activating neurons inhibit surrounding neurons (null domain inhibition). Neurons with a null domain inhibition effect should be given higher priority in visual processing. The lower the energy, the more the neuron t is distinguished from the surrounding neurons and the higher the importance. The neuron minimum energy function is defined as Eq (4).


$$\begin{array}{c} e_{k}^{*}=\frac{4\sigma^{\text{2}}+\lambda }{t_{k}-{\overset{―}{u}}^{\text{2}}+2\sigma^{\text{2}}+2\lambda } \end{array}$$ (4)

where λ is the regularity term, t_k_ is the k^th^ neuron of the input feature map on a single channel, u is the mean of all neurons on a single channel, and σ^2^ is the variance of all neurons on a single channel.

The SimAM is placed at three key locations in the FAnet framework: (1) immediately after the C2Star module in the backbone, enhancing low level texture extraction; (2) at the output of the Feature Pyramid Network (FPN), optimising multi-scale feature fusion; and (3) at the segmentation header input, improving mask generation quality.

### Multi-scale dynamic fitting module

To precisely calculate FA peel thickness, the multi-scale dynamic fitting module is integrated into the FAnet framework. Initially, the pixel areas representing the inner (flesh) and outer (peel) regions of FA fruits are extracted using the YOLOv8n-seg model [27]. Subsequently, the peel thickness is derived by calculating half the difference between the inner and outer diameters. To ensure accurate mapping from pixel based measurements to real world dimensions, a multi-scale dynamic fitting approach is utilized.

I. Relationship between target area and actual diameter

Specifically, FA fruit cross sectional areas are classified into inner and outer diameter regions, representing the flesh and outer peel, respectively. To enhance model stability and accuracy, pixel area data is standardized and normalized to a range of [0, 1] using Eq (5), preventing large eigenvalues from adversely affecting polynomial regression. After normalization, polynomial regression is applied to the normalized pixel areas (a_inner and a_outer). This enables accurate prediction of the actual diameter (D), as shown in the Eq (6).


$$\begin{array}{c} A_{norm}=\frac{A-A_{min}}{A_{max}-A_{min}} \end{array}$$ (5)

Where A_min_ and A_max_ are the minimum and maximum areas of all training samples.

The pixel areas of the inner and outer diameters of FA were extracted by the YOLOv8-seg model and normalized using Eq (5) to obtain a and a for polynomial regression in order to obtain the predicted actual diameter D. The calculation process is shown in Eq (6).


$$\begin{array}{c} \begin{array}{c} D_{inner}=f\left(A_{inner}^{pixel}\right)=a_{n}(A_{inner}^{pixel}{)}^{n}+a_{n-1}(A_{inner}^{pixel}{)}^{n-1}+\cdots a_{\text{1}}(A_{inner}^{pixel}{)}^{\text{1}}+a_{\text{0}}\\ D_{outer}=g\left(A_{outer}^{pixel}\right)=b_{n}(A_{outer}^{pixel}{)}^{n}+b_{n-1}(A_{outer}^{pixel}{)}^{n-1}+\cdots b_{\text{1}}(A_{outer}^{pixel}{)}^{\text{1}}+b_{\text{0}} \end{array} \end{array}$$ (6)

Where a_n_, a_n-1_,..., a_0_ and b_n_, b_n-1_,..., b_0_ are the fitted coefficients.

Subsequently, the peel thickness is determined based on Eq (7).


$$\begin{array}{c} t_{peel}=\frac{D_{outer}-D_{inner}}{\text{2}} \end{array}$$ (7)

Considering that peel thickness is derived from both outer and inner diameter predictions, estimation errors may propagate according to Eq (8).


$$\begin{array}{c} \Delta T=\frac{\Delta OD-\Delta ID}{\text{2}} \end{array}$$ (8)

where ΔT denotes thickness error caused by prediction deviations in OD and ID. This relationship was further evaluated through threshold-sensitivity analysis in the grading experiments.

To ensure reproducibility, the regression procedure in MSDFM is described in detail as follows. The normalized pixel area is defined as the ratio between the segmented region area and the total image area, which reduces the influence of scale variations. Separate polynomial regression models are trained for the estimation of inner diameter and outer diameter.

In this study, a second-order (quadratic) polynomial regression is adopted, which provides a balance between fitting accuracy and model simplicity. The dataset used for regression is divided into training and testing subsets with a ratio of 8:2. During training, a five-fold cross-validation strategy is applied to improve robustness, and the final model parameters are selected based on the average validation performance.

To prevent overfitting, the fitting performance is evaluated using the coefficient of determination (R²) and residual analysis. Models with unstable fitting behavior are discarded. In addition, for targets of different size ranges, scale-specific regression functions are selected based on empirical error minimization, enabling more accurate mapping across multiple scales.

II. Quality grading

The Chinese group standard “Commodity specification grade of Chinese herbal medicines - Aurantii Fructus” (T/CACM 1021.30–2018) stipulates the commodity specification grade of Aurantii Fructus. The grading is based on the actual diameter D of FA shells and with the peel thickness, as shown in **Table 2**.

**Table 2:** FA grading standard.

Quality Grade	Outer Diameter Range (cm)	peel Thickness Range (cm)	Criteria
Grade 1	3 < outer < 5	0.6 ≤ peel ≤ 1.3	Must satisfy **both** outer diameter and thickness requirements
Grade 2	3 < outer< 5	0.4 ≤ peel < 0.6	Must satisfy **both** outer diameter and thickness requirements
Grade 3	outer ≤ 3 **or** outer ≥ 5	peel < 0.4 **or** peel > 1.3	Satisfy **at least one** of the following: (1) Out-of-range diameter (2) Out-of-range thickness

Additionally, different regression formulas tailored to distinct object scales are employed, as outlined in Eq (9), ensuring optimal multi-scale fitting performance.


$$\begin{array}{c} D_{predicted,scale}=a_{0,scale}+a_{1,scale}A_{input,scale}+a_{2,scale}A_{input,scale}^{\text{2}} \end{array}$$ (9)

Where A_input, scale_ is the pixel area of the target at that scale, D_predicted, scale_ is the predicted diameter of the target based on the pixel area at that scale, and a_0, scale_, a_1,, scale_ and a_2,scale_ are the fitting coefficients at that scale.

### Summary of architectural modifications

For clarity, all modifications are summarized in **Table 3**.

**Table 3:** Final architecture configuration of FAnet.

Stage	Component	Modification
Backbone	C2f	Replaced by C2Star
Backbone	Feature output	SimAM inserted after C2Star
Neck (FPN)	Feature fusion	SimAM inserted after FPN
Head (Seg)	Segmentation	SimAM inserted before seg head
Post-process	MSDFM	Added for geometric fitting

All descriptions in this manuscript refer to this unified architecture.

## Experiment

These procedures ensured that reported segmentation and grading performance were evaluated under a leakage-controlled experimental setting.

### Experimental environment

In this research, the FAnet model was trained on a workstation equipped with a GeForce RTX 3090TI GPU and an Intel i7-14700HX processor. The programming language was Python 3.8 and the deep learning models were developed using the PyTorch framework version 1.12.0. Performance tests and evaluations were conducted on a portable device with a GeForce RTX 2060 GPU, 32 GB RAM, and an Intel(R) Core(TM) i7-1165G7 processor at 2.80 GHz.

### Ablation experiment

Ablation experiments conducted on the FA datasets (**Table 4**) revealed that each module contributed specific advantages. Specifically, the C2Star is introduced alone. It optimizes the model by implicitly stacking features. As a result, the precision improves by 3.61 percentage points (pp). The recall improves by 15.0 pp. And the mAP@50 improves by 5.32 pp. Meanwhile, the model params are reduced by 7.1%. The computational complexity (GFLOPs) is reduced by 6.7%. This indicates that the C2Star effectively eliminates computational redundancy. It also enhances boundary feature extraction.

**Table 4:** Ablation experiment result.

YOLOv8n-seg	C2Star	SimAM	Precision (%)	Recall (%)	mAP@50 (%)	mAP@95 (%)	Params (M)	GFLOPs (G)
√	×	×	88.82	80.75	92.19	56.11	3.26	8.9
√	√	×	92.43	95.73	97.51	59.99	3.03	8.3
√	×	√	94.62	98.18	98.50	64.08	3.26	8.6
√	√	√	96.12	98.77	98.60	77.08	2.97	7.9

When introduced alone, the SimAM’s channel space attention mechanism has notable effects. It improves recall by 98.18% (17.43 pp up from baseline) and mAP@95 by 64.08% (7.97 pp up from baseline), and cuts GFLOPs to 8.6. This shows the SimAM boosts edge contour recognition rate via parameter free attention and slightly reduces computational cost.

When C2Star and SimAM were used together, precision, recall, and mAP@95 reached 96.12%, 98.77%, and 77.08%, respectively. Compared with the better single-module result, the combined model increased precision by 1.50 pp, recall by 0.59 pp, and mAP@95 by 13.00 pp. It also reduced the number of parameters to 2.97 M and GFLOPs to 7.9. These results suggest that the two modules are complementary: C2Star improves semantic consistency through cross-layer feature reuse, whereas SimAM further refines the discriminative distribution of fused features.

Further analysis of the feature maps (Fig 6) confirms these results. In the baseline YOLOv8n-seg model, the feature extraction are rather scattered and lacked adequate description of key target details such as contours and internal structures. With C2Star integration, the feature extraction concentrates more effectively on key structural regions, enhancing the extraction of detailed structural information. With the addition of the SimAM, the characteristics of the target region are improved and the background interference is effectively suppressed. Combining C2Star with SimAM clearly defines the target structure, improves feature focusing, and generates a comprehensive and detailed feature map. Consequently, experiments show that the improvement optimises the quality of feature representation in the baseline model. It also lays the foundation for better quality features for the accurate execution of subsequent segmentation tasks.

**Fig 6:**
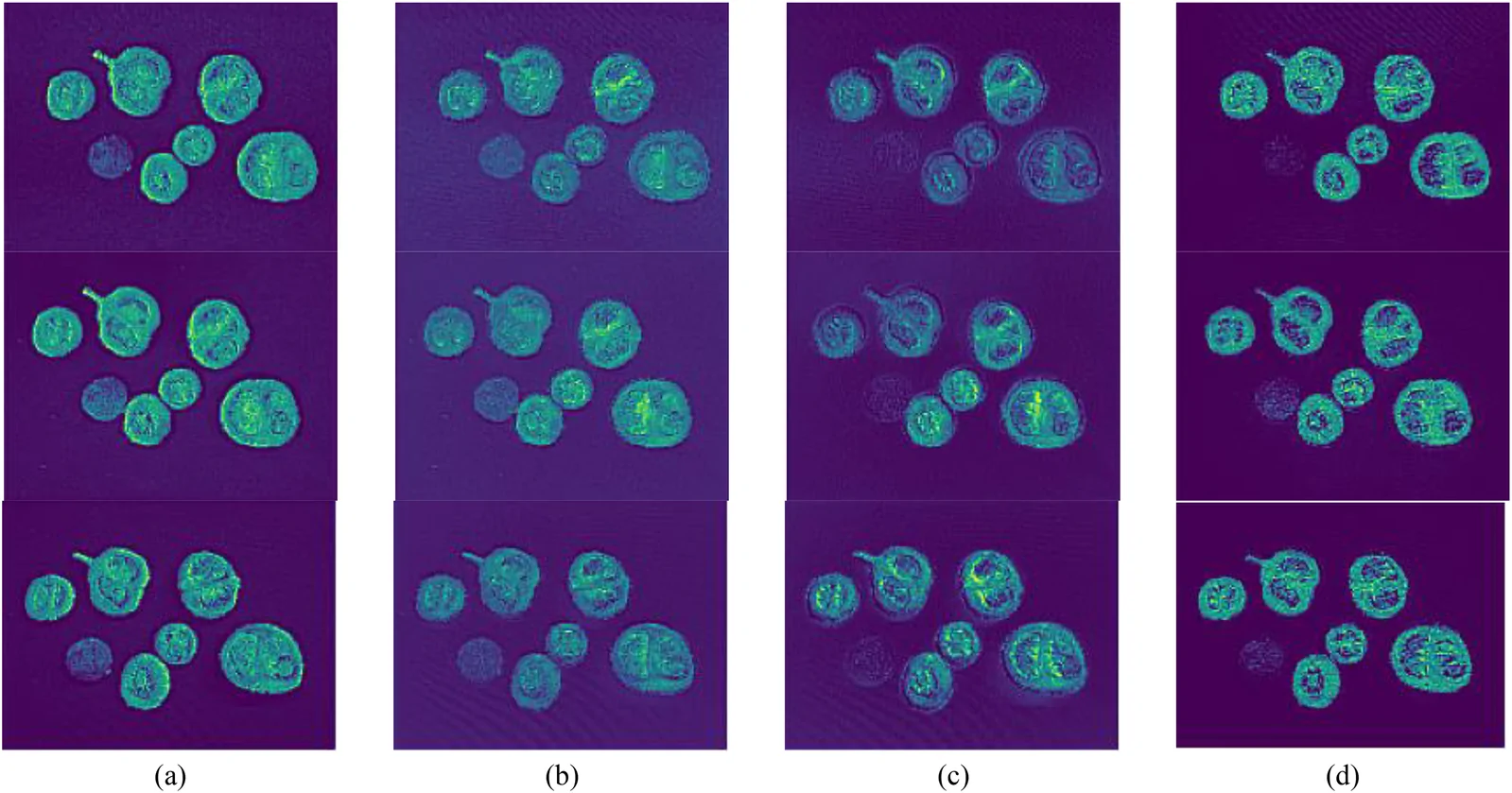
Feature map comparison: (a) baseline model; (b) add C2Star; (c) add SimAM; (d) add C2Star and SimAM.

Fig 7 presents the recognition results and confidence distribution of the model labels for “Fructus Aurantii” and its internal “locule”, with 6 typical samples selected (each containing multiple FA individuals). For most samples, the recognition confidence of Fructus Aurantii main bodies stably ranged within the high interval of 0.92–0.97. Specifically, the maximum confidence of Fructus Aurantii in Fig 7(c) reached 0.93, that in Fig 7(d) hit 0.97 (the highest value across all 6 samples), and the maximum confidence in both Fig 7(b) and Fig 7(f) was 0.96. All these fully verify the excellent recognition stability of the model for intact and clear Fructus Aurantii samples. Only Fig 7(a) showed a low-confidence exception: one individual of Fructus Aurantii in this sample had a confidence as low as 0.78. This sample had a dark overall tone and blurred edge contours, which likely reduced the amount of effective feature information available to the model. The confidence scores of the internal locules generally followed those of the corresponding FA main bodies. For samples with high confidence in Fructus Aurantii main bodies, the recognition confidence of internal locules was synchronously high. For instance, the maximum confidence of 0.93 for Fructus Aurantii in Fig 7(c) corresponded to locule confidences of 0.92 and 0.94. The maximum confidence of 0.97 in Fig 7(d) matched a locule confidence of 0.92. The maximum confidence of 0.96 in both Fig 7(b) and Fig 7(f) corresponded to locule confidences of 0.90 and 0.92, respectively. In contrast, when the confidence of Fructus Aurantii main bodies was low, the confidence of locules decreased accordingly. This regularity clearly reflects the critical supporting effect of the feature integrity of Fructus Aurantii main bodies on the recognition of their internal structures. It also provides intuitive and reliable references for the model performance under non-ideal imaging conditions such as insufficient illumination.

**Fig 7:**
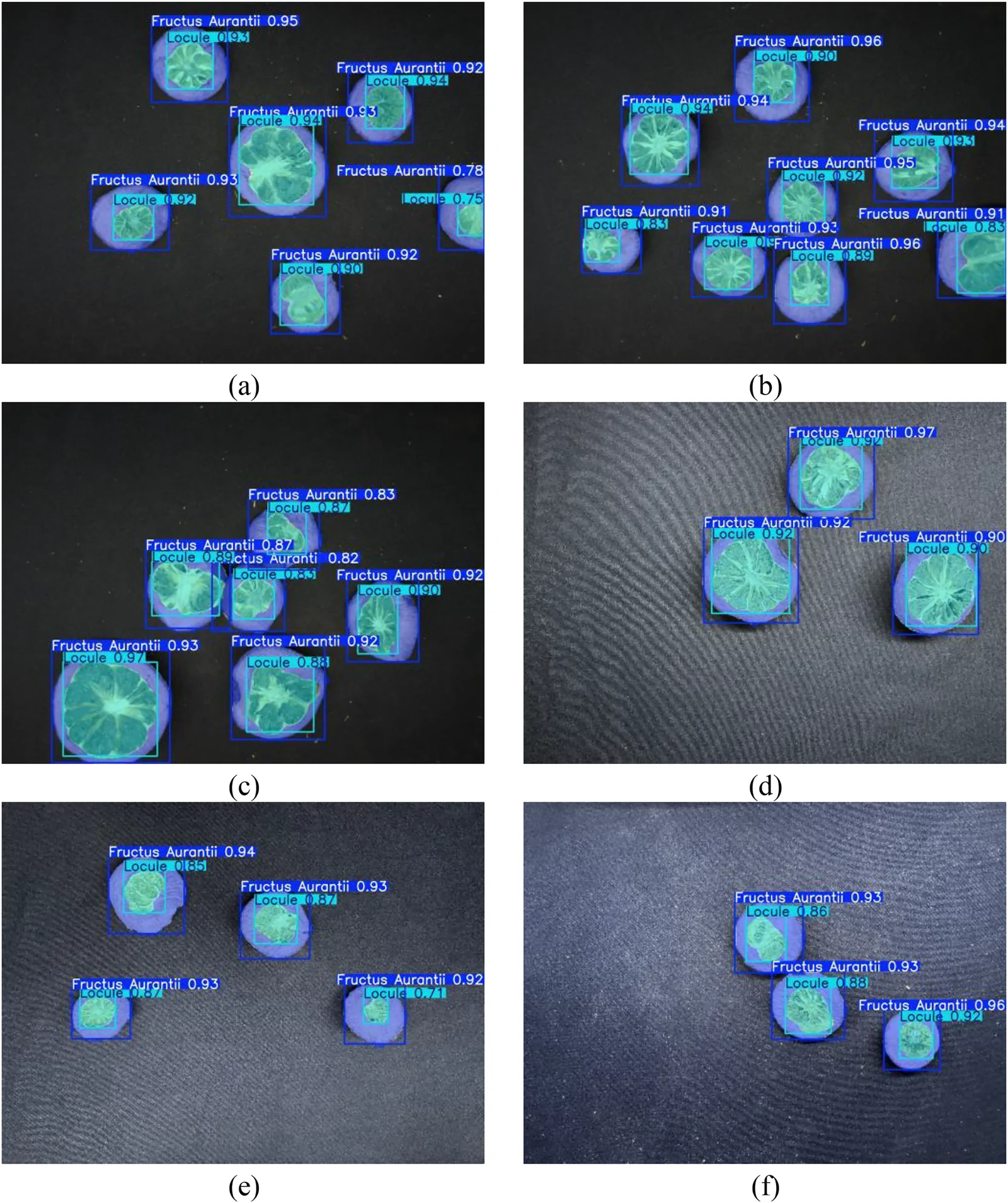
Recognition results and confidence distribution of the FANet for Fructus Aurantii and its internal Locule.

### Algorithm comparison experiment

This research systematically evaluates the performance advantages of FAnet in the instance segmentation tasks by comparing it with mainstream segmentation models (**Table 5**). All comparison models were trained and evaluated under fully comparable conditions, including the same input image resolution, identical train/validation/test split, the same augmentation policy, the same epoch budget, the same optimizer settings, and comparable hyperparameter-tuning effort. This protocol was used to ensure fairness of model comparisons. After integrating the C2Star and SimAM, the FAnet achieves 96.12% precision and 98.77% recall. This is 7.3% and 18.02% higher than the second ranked RTMDet model. The C2Star effectively captures multi-scale contextual information through implicit feature stacking. The SimAM optimises the spatial channel feature distribution through cross dimensional interactions, thus reducing both missed detections (FN) and false detections (FP) of complex feature FA.

**Table 5:** Performance comparison of mainstream lightweight segmentation models and FAnet.

Model	Precision (%)	Recall(%)	mAP@50(%)	mAP@95(%)	Params(M)	GFLOPs
U-Net++	86.25	78.35	90.21	54.99	9.04	16.6
RTMDet	88.82	80.75	92.19	56.11	5.62	11.8
YOLO v5n seg	82.19	74.36	86.35	51.47	1.77	4.7
YOLOv8n-seg	84.57	76.12	88.62	52.83	3.26	8.9
FAnet(Ours)	96.12	98.77	98.60	77.08	2.97	7.9

Furthermore, the key evaluation metrics demonstrate FAnet superior performance. Compared to the baseline model, FAnet’s mAP@50 and mAP@95 metrics improved by 9.98% and 24.25%, respectively. The results show that the exponentially expanding implicit dimensions of the C2Star module are highly effective in accurately delineating target edges. While maintaining the lightweight nature of the YOLO models, FAnet achieves an optimal balance between model complexity and computational efficiency, with a parameter reduction of 8.9% (2.97 M) and GFLOPs optimized at 7.9. However, parameters and GFLOPs are only indirect indicators of computational efficiency and do not alone establish hardware-level deployment performance.

To further assess robustness beyond single-run point estimates, an uncertainty analysis based on random-seed sensitivity was conducted (**Table 6**). Repeated training under different initialization seeds produced only minor metric variation, indicating that the observed performance improvements of FAnet were stable rather than seed-dependent.

**Table 6:** Random-seed sensitivity analysis of FAnet performance (n = 3).

Random Seed	Precision(%)	Recall(%)	mAP@50(%)
Seed 1	96.8	98.7	98.1
Seed 2	96.5	98.4	98.8
Seed 3	96.9	98.6	98.0
Mean ± SD	96.7 ± 0.2	98.6 ± 0.2	98.0 ± 0.2

Note: Variation across random seeds was limited, supporting training stability.

As depicted in Fig 8, the segmentation model comparison results highlight the capabilities of FAnet. The YOLOv5n-seg model suffers from missed detections and fails to accurately identify the pith region. The YOLOv8n-seg model achieves basic segmentation but inadequately details flesh textures. The U-Net++ model, despite having a powerful encoder decoder architecture, struggles with edge localisation. The RTMDet model is primarily a detection oriented model that falls short in the accuracy of segmentation details. Conversely, the FAnet model showed stronger segmentation performance under the evaluated conditions. It integrates hierarchical feature fusion through the C2Star structure. At the same time, it emphasizes targeted regions via SimAM. As a result, segmentation accuracy was consistently improved under the evaluated conditions. The model can clearly delineate target boundaries and complex textures. It can also substantially reduce segmentation error. This supports the effectiveness of the integrated model design for the evaluated task. The performance gains of FAnet exceeded the observed random-seed variability, suggesting that the reported improvements are attributable to architectural changes rather than stochastic training effects.

**Fig 8:**
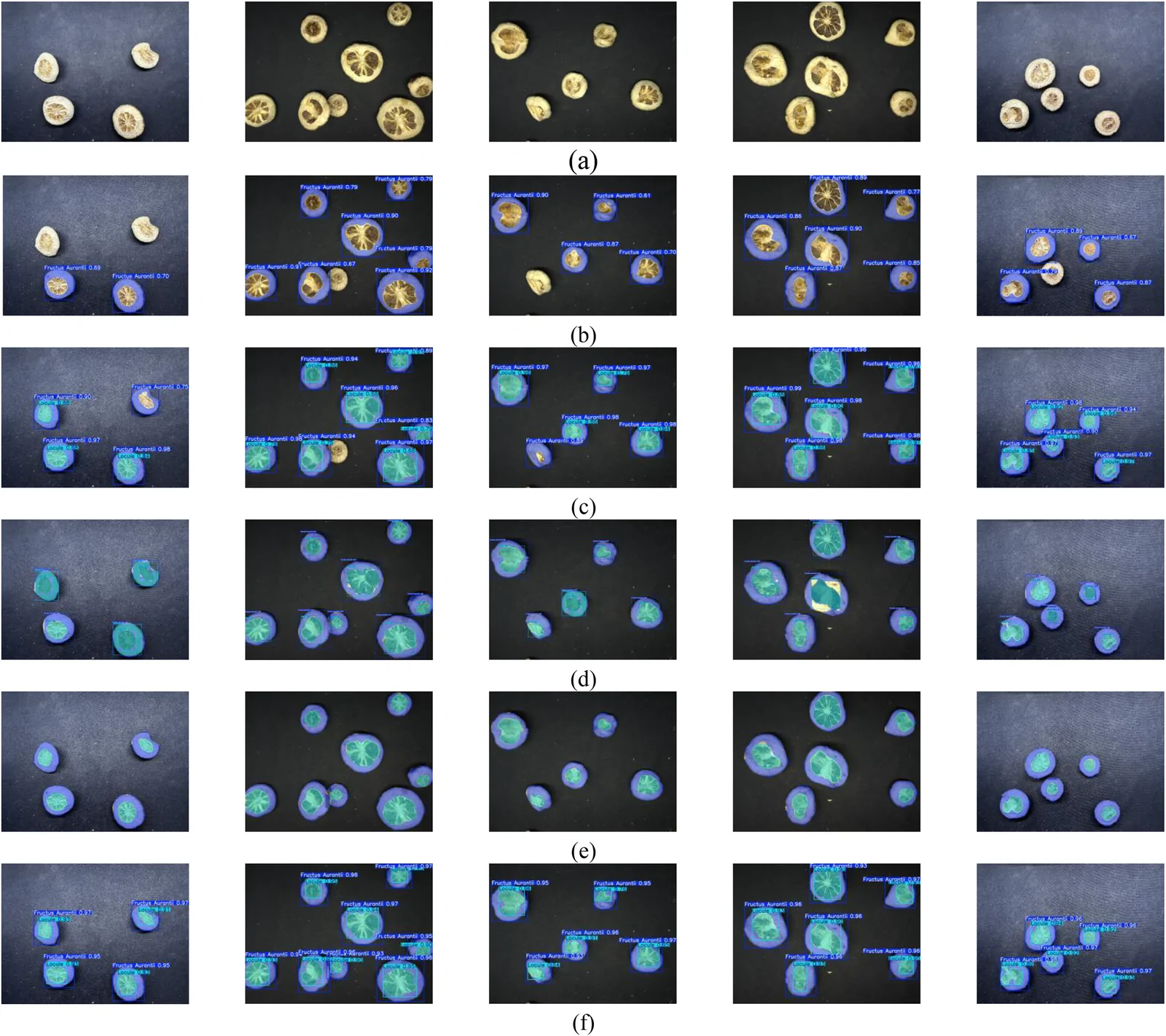
Algorithm comparison result: (a) Original image; (b) YOLO v5n seg; (c)YOLOv8n-seg; (d) Unet++; (e) RTMDet; (f) FAnet.

### Graded experiment

A total of 35 samples were included in the grading validation experiment, representing the full independent grading test set rather than an illustrative subset. All available samples with manually measured OD, ID, and peel thickness reference values were used for grading validation. The grading set covered all three grades and included boundary-sensitive samples relevant to threshold-based classification.

In the FA grading experiment, the grading standard based on ID, OD, and peel thickness was validated through systematic measurement and manual labeling. As shown in Fig 9, the FAnet predicted FA’s ID, OD and calculated the peel thickness. Evaluation grades were then given based on the grading standard. Each predicted image was labeled with the OD, ID, peel thickness, and the grading was determined according to the double threshold condition described in Section 3.3. The majority of the sample’s demonstrated the rigour of this grading system. For example, a FA with an OD of 4.40 cm, an ID of 2.88 cm, and a thickness of 0.76 cm met both Grade 1 conditions (thickness between 0.6 and 1.3 cm and OD between 3 and 5 cm). Conversely, a FA with an OD of 2.75 cm, ID of 1.61 cm, and thickness of 0.57 cm satisfied the thickness range for Grade 2 but was categorized as Grade 3 due to falling outside the OD threshold. This supports the feasibility of the proposed grading rules, although uncertainty remains near threshold boundaries. However, in order to reduce manual errors and improve efficiency, it is necessary to automate peel thickness calculation and grading through model based post processing.

**Fig 9:**
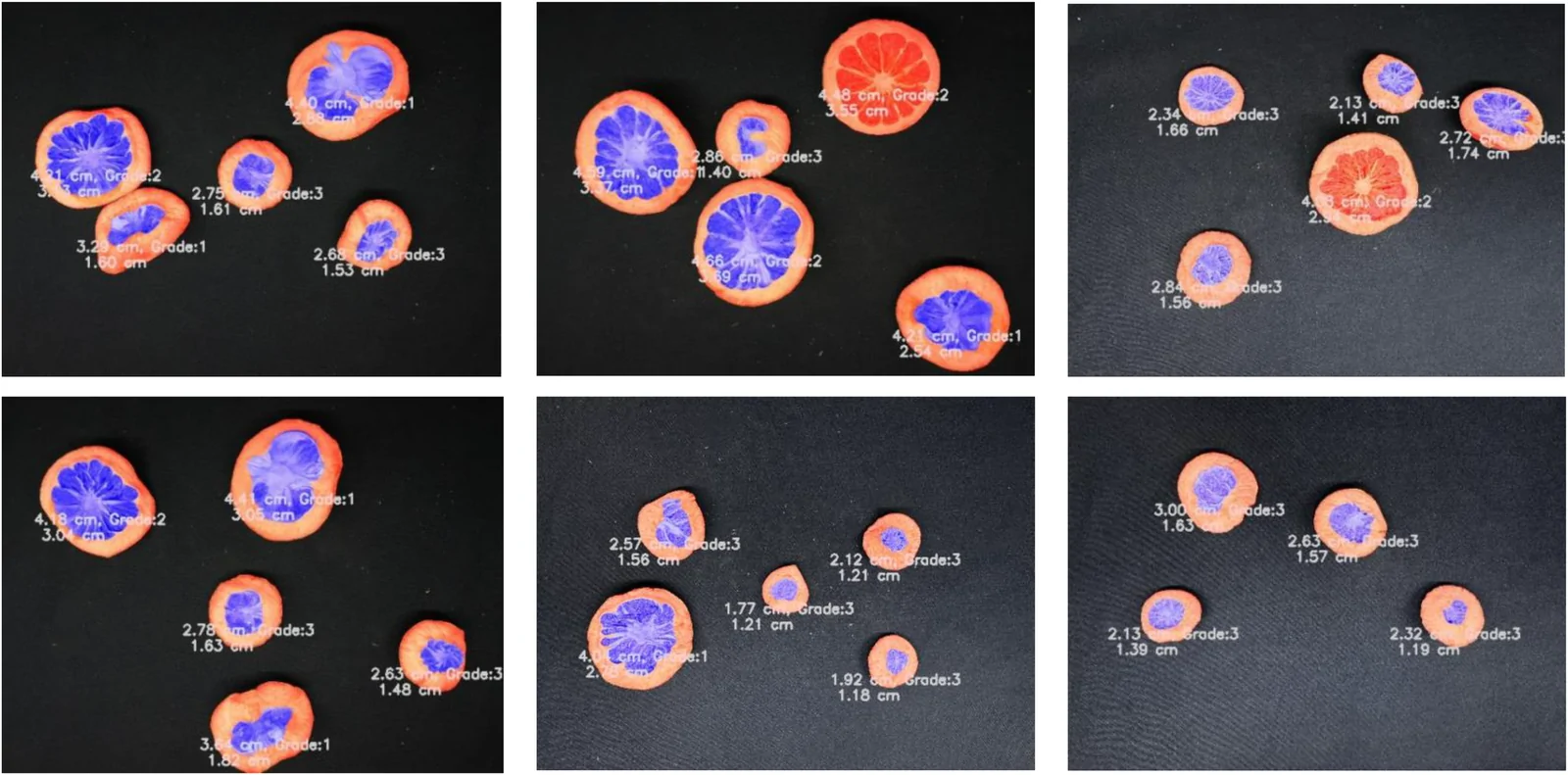
The grading experiment result.

In addition, analyzing the prediction accuracy of the inner diameter, outer diameter, and peel thickness. The results showed significant differences in the prediction of fruit flesh and peel (Fig 10). The model predicted the outer diameter very well. Where the evaluation result R² was 0.989, RMSE was 0.091 cm and rRMSE was 2.9%, indicating that the predictions were highly consistent with the measured values. This may be due to the uniform structure of the peel and the well defined edge portion. In contrast, the accuracy of inner diameter prediction was slightly lower. The evaluation result R² was 0.943 with a RMSE of 0.185 cm and an rRMSE of 9.1%. The increase in error may be due to the irregularity of the flesh portion of the fruit causing difficulty in identification.

**Fig 10:**
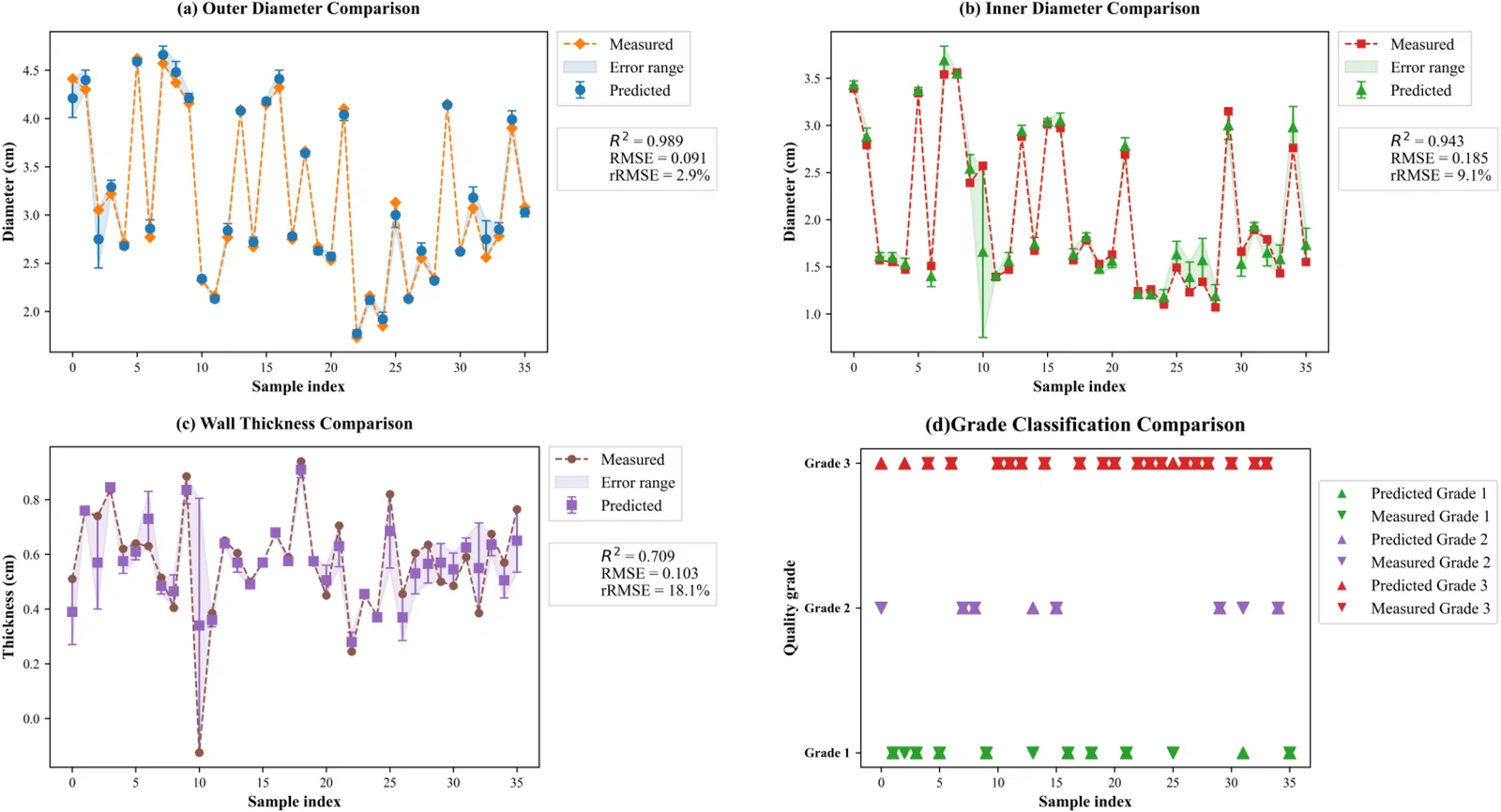
Real and predicted values comparison result: (a)outer diameter; (b) Inner diameter; (c) thickness; (d) grade classification.

Peel thickness, calculated from predicted OD and ID, exhibited the lowest accuracy among the three evaluated traits, with an R² of 0.709, RMSE of 0.103 cm, and rRMSE of 18.1%, indicating that grading uncertainty is primarily associated with the derived thickness variable rather than with direct diameter estimation. Because peel thickness is computed from both OD and ID predictions, prediction deviations may accumulate and become amplified, particularly near grade decision thresholds.

Among the 35 graded samples, 5 were misclassified, corresponding to an overall grading accuracy of 85.7%. Misclassifications occurred mainly near threshold boundaries, where small deviations in OD or ID caused shifts in final grade assignment.

Direct grade-level evaluation results are presented in Table 7 and Fig 11. The confusion matrix showed that most classification errors occurred between adjacent grades, whereas no direct misclassification between Grade 1 and Grade 3 was observed. This indicates that grading errors were mainly associated with threshold-sensitive boundary samples rather than systematic model failure.

**Table 7:** Grade-level classification performance of FAnet.

Grade	Precision(%)	Recall(%)	F1-score (%)
Grade 1	83.3	90.9	87.0
Grade 2	80.0	72.7	76.2
Grade 3	92.3	92.3	92.3

Note: The overall grading accuracy was 85.7% (30/35).

**Fig 11:**
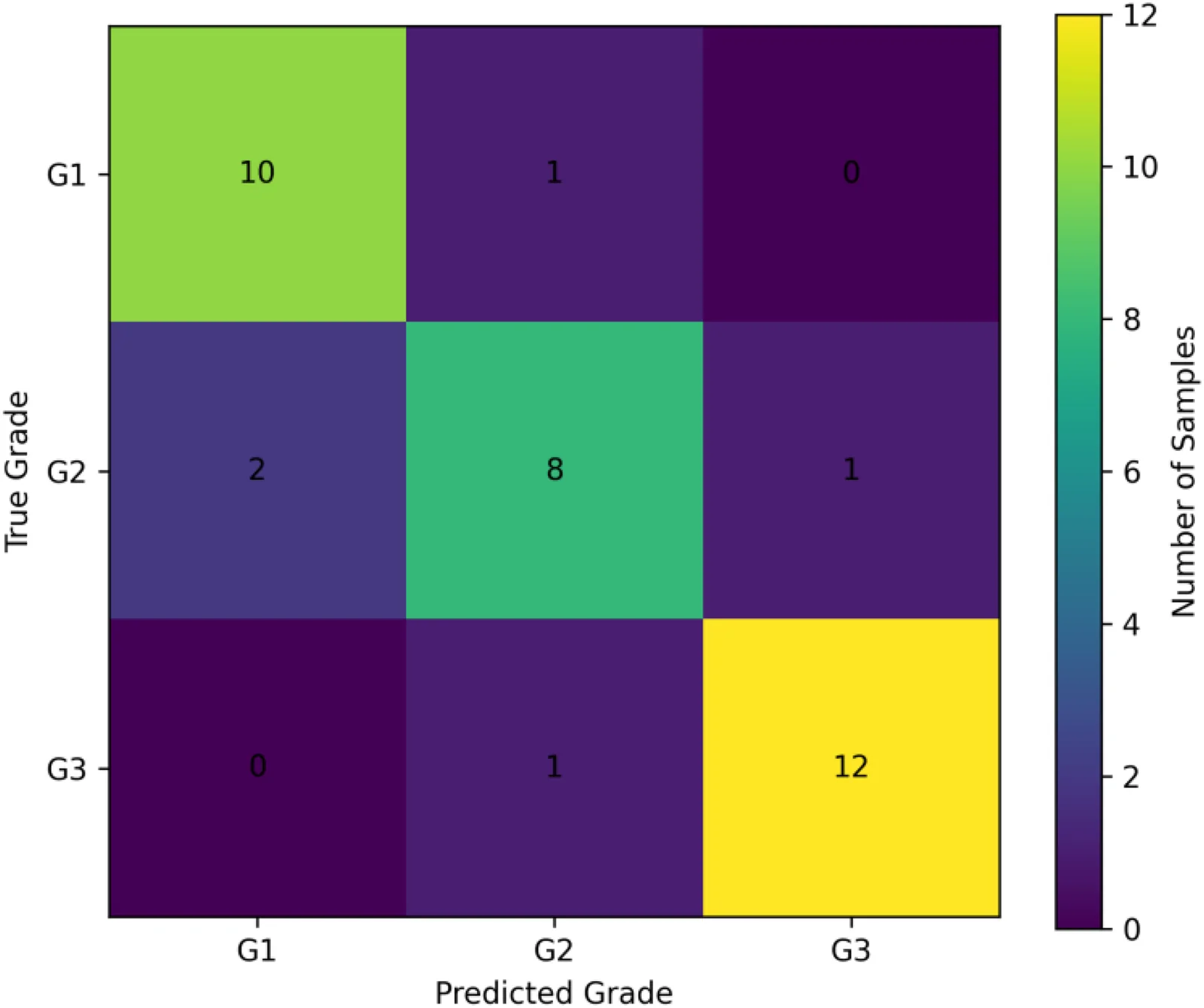
Confusion matrix of quality grading results obtained using FAnet across 35 test samples.

To further assess robustness, a sensitivity analysis was conducted by introducing simulated OD and ID perturbations (Table 8). Grading stability decreased from 85.7% under baseline conditions to 80.0% and 71.4% under ±2.5% and ±5.0% perturbations, respectively. Among all categories, Grade 2 exhibited the highest sensitivity, indicating that samples located near grading thresholds were more susceptible to propagated measurement errors.

**Table 8:** Sensitivity analysis of grading stability under simulated OD and ID perturbations.

Panel A. Overall stability under perturbation			
Perturbation Level	Stable Sample	Grade Changes	Stability (%)
0% (Baseline)	30	5	85.7
±2.5%	28	7	80.0
±5.0%	25	10	71.4
**Panel B. Grade-specific stability under ±5% perturbation**			
Grade	Stability (%)		
Grade1	90.9		
Grade2	63.6		
Grade3	84.6		

Note: Simulated perturbations were introduced to evaluate the propagation of OD/ID prediction deviations into final grade assignments. Reduced stability was mainly observed in boundary-sensitive samples.

These findings suggest that the proposed framework demonstrates the feasibility of automated grading, while further improvement in peel-thickness estimation is still needed for robust deployment.

The sensitivity analysis further suggests that current grading limitations arise primarily from threshold effects in derived peel-thickness estimation, rather than from instability in the segmentation framework itself. This distinction indicates that future improvements may be achieved through refinement of thickness measurement or adaptive threshold strategies, without requiring fundamental changes to the segmentation architecture.

It should be noted that the 35 samples used in the grading experiment represent the complete independent validation set available under the present measurement conditions, rather than a selected subset. Although this sample size remains limited for establishing deployment-level robustness, the inclusion of all available samples and the presence of boundary-sensitive cases support the validity of the present feasibility analysis.

Although all available independent grading validation samples were included in the present study, the sample size remains limited for fully establishing deployment-level robustness. Future work will expand grading validation across larger sample populations, additional cultivars, and broader threshold conditions to further assess generalizability.

## Conclusions

(1) The proposed FAnet framework demonstrates strong segmentation performance and preliminary feasibility for automated quality grading of Fructus Aurantii. While outer-diameter prediction achieved high accuracy, grading reliability remains constrained by uncertainty in peel-thickness estimation, particularly near threshold boundaries. Furthermore, the present grading validation was conducted using the full available independent test set of 35 samples, which supports feasibility assessment but remains limited for establishing deployment-level robustness. Therefore, the present study supports the feasibility rather than definitive robustness of practical grading, and further refinement of thickness measurement together with expanded validation is required before deployment-oriented applications.
(2) The present research has three major limitations that need to be addressed. First, model validation is restricted to a single variety of Fructus Aurantii, and its generalizability to other herbal varieties (e.g., Chenpi and Qingpi) remains unconfirmed. Second, although the lightweight architecture of FAnet suggests potential suitability for future implementation on mobile or edge devices, deployment capability has not yet been validated through hardware-level benchmarking. In particular, actual inference speed, memory usage, and end-to-end runtime have not been evaluated on target hardware such as Raspberry Pi. Therefore, the present study supports deployment-oriented feasibility rather than verified hardware-level deployment performance. Third, the MSDFM is developed under controlled imaging conditions, with all images acquired at a fixed distance. Therefore, variations in imaging distance and viewpoint are not explicitly modeled. Future work will explore more robust geometric modeling strategies to improve adaptability under diverse imaging conditions. Finally, the indirect method adopted in Fructus Aurantii grading experiments to calculate peel thickness introduces inherent bias, which may lead to erroneous grade predictions especially when the fruit flesh is irregularly shaped. Additionally, the use of fixed grading thresholds increases the risk of classification errors near threshold boundaries.
(3) To address the above limitations, the following targeted research directions are proposed. First, incorporate hyperspectral imaging or laser ranging technology. This technology can realize direct, non destructive measurement of peel thickness. In this way, it can eliminate the bias caused by indirect calculation methods. Second, develop a dynamic threshold adjustment mechanism. Base it on fuzzy logic or other probabilistic models. Integrate confidence scores. This will enhance the flexibility and robustness of the grading system. It’s especially beneficial for samples near threshold values. Although leakage-control and annotation quality-control procedures were implemented to improve reliability, future work may further strengthen annotation consistency through multi-annotator agreement analysis and expanded validation across larger datasets. Finally, expand the validation dataset. Include multiple varieties of citrus related herbs like Chenpi and Qingpi. This is to verify the cross variety generalizability of the model. Optimize the model for future edge-oriented implementation using techniques such as model quantization and hardware acceleration, and conduct hardware-level timing validation on edge devices such as Raspberry Pi.
